# Francis Sibson (1814–1876): A serial specialist

**DOI:** 10.1177/09677720251316348

**Published:** 2025-04-28

**Authors:** Henry Connor

**Affiliations:** Honorary Research Fellow in the History of Medicine, 1724 University of Birmingham, Hereford, UK

**Keywords:** 19th century, anaesthesia, infectious diseases, vivisection, cardio-respiratory, medical societies and medical politics, disease classification, medical equipment, curare

## Abstract

In a diverse career Sibson performed some of the earliest saline infusions for cholera, some of the first experiments with curare and its possible use in rabies and he was among the first British authors to distinguish between typhoid and typhus. He published on respiratory physiology and mechanisms of respiration and on the anatomy of the chest and abdominal viscera including the changes caused by movement and disease. He investigated the use of ether and chloroform to treat neuralgia, probably practised surgical anaesthesia and sat on a commission investigating the safety of chloroform. His interests later changed to cardiac disease, especially pericarditis, endocarditis and aortic aneurysms. His membership of medical societies led to an involvement in medical politics, such as the conditions of service of army and poor law doctors, the sale of arsenic, reform of the membership of the General Medical Council and *The Nomenclature of Disease* which was an early and very successful attempt at the classification of diseases. He was invited to give evidence to a Royal Commission on vivisection and sat on several government committees and enquiries including the siting and design of infectious diseases and Poor Law hospitals and the Metropolitan District Asylums Board.

## Biographical background

### Early life and career: Maryport and Edinburgh

Sibson was born near Maryport in what was then Cumberland, now Cumbria, where his father was an unsuccessful farmer.^1^ The family then moved to Edinburgh where he lost his mother and three of his four brothers within just a few years.^2^ His surviving brother, Thomas, was a significant artist who provided illustrations for the works of Dickens and other authors. He died from tuberculosis in tragic circumstances when Francis was thirty.^1^ Francis was called upon to support his father and brother from an early period.^2^

At the age of 14 Francis was apprenticed to Professor John Lizars (1791/1792–1860), anatomist and surgeon in Edinburgh. It was Lizars’ influence which triggered Sibson's interest in anatomical research though in character the two men were very different: Sibson friendly and conciliatory^2^ whereas Lizars was distinctly litigious.^3^ Sibson passed the Edinburgh LRCS in 1831 at the age of only 17, his age being registered without date of birth as 18, a procedure which was common at the time.^2^ He subsequently went into general practice at Cockernouth near where he was born, but soon moved to the pathology department at Guy's Hospital in London where the physician and pathologist Thomas Hodgkin (1798–1866) became his mentor and friend.^2^ Hodgkin subsequently praised Sibson's work as a practical anatomist and physiologist.^4,5^ He was also instrumental in helping Sibson to obtain his next post in Nottingham and thereafter for introducing him to London practice.^6^

### The Nottingham years 1835–1848

In 1835, having obtained his Licentiate of the Society of Apothecaries, Sibson took up the post of Resident Surgeon and Apothecary at the Nottingham General Hospital. His clinical practice led to publications on typhoid and its distinction from typhus, on the use of wourali (curare) in the treatment of rabies (hydrophobia) and on the use of the newly described inhalational anaesthetic agents for treatment of facial neuralgia.

In addition to his clinical work he found time for research. The latter included anatomy, cardiac and respiratory physiology and medicine which he continued after he left Nottingham for London and which will be discussed later. The respiratory physiology was to prove useful in his research with inhalational anaesthetic agents but it was not until much later that his description of the thickened supraclavicular fascia, subsequently named Sibson's fascia by others, became relevant to the practice of supraclavicular brachial plexus block using local anaesthesia.^7^

### The move to London 1848–1876

By 1848 Sibson had decided to follow advice from friends that a move to London would provide ‘a wider range for the development of his special talents’.^2^ Although others urged him to remain in Nottingham he decided to make the move but resolved to practice in London as a physician and not, as he had in Nottingham, as a surgeon. He was able to contemplate this change because friends promised to support him financially until he was established. To practice as a physician also required that he pass the University of London's MB and MD examinations. He passed the former with honours and the latter with a gold medal just one week later.^2,8^ He passed the MRCP (Lond) examination and was elected a Fellow of the Royal Society in 1849 and FRCP (Lond) in 1853.

Sibson would appear to have lived in at least three different houses in Brook Street but further investigation revealed that he had moved houses only once within this street and that the other numbers were due to renumbering of the street.^9^ In 1849, he gave a very successful series of evening lectures on visceral anatomy at his home. According to one of his biographers these lectures helped to establish his reputation.^2^ They may also have helped to expedite his election to the Royal Society as ‘Distinguished in his acquaintance with the science of Physiology’ because, just one year after his arrival in London, those who were already able to nominate him ‘From personal knowledge’ included: Sir Benjamin Brodie (1783–1862), surgeon and physiologist; George Newport (1803–1854), surgeon and entomologist; Sir Erasmus Wilson (1809–1884), dermatologist and philanthropist; Professor Charles James Blasius Williams (1805 −1889), chest physician; Richard Bright (1789–1858), physician; Sir John Forbes (1787–1861), physician and medical editor; William Sharpey (1802–1880), physiologist; Francis Kiernan (1800–1874), anatomist and surgeon; Alfred Swaine Taylor (1806–1880), medical jurist and toxicologist; Joseph Toynbee (1815–1866), ear surgeon; Charles Brooke (1804–1879), surgeon and inventor of measuring instruments; William Baly (1814–1861), physician; John Edward Gray (1800–1875), zoologist and museum curator; George Owen Rees (1813–1889 physician; and George Gulliver (1804–1882), surgeon, anatomist and physiologist.^10^ When St Mary's Hospital opened in 1851, he was appointed one of its first physicians, retiring to its consulting staff in 1871 after serving the prescribed twenty years. Meanwhile he lectured on medicine at the Grosvenor Place School of Medicine, and at Samuel Lane's School of Anatomy, also in Grosvenor Place.^2,8^ Finally he was appointed lecturer in medicine at St Mary's Hospital Medical School when it opened in 1854.^2^ He was a popular and inspirational teacher and an accurate diagnostician but ‘showed little interest in therapeutics, uniformly prescribing tincture of perchloride of iron regardless of his own precise and painstaking diagnosis’.^8^

In 1858 he married Sarah Mary Ouvry (1822–1898). She came from a Huguenot family which had arrived in England in 1683 and which had become one of the largest silk weaving families in London. Her brother Frederic (1814–1881) was a lawyer and antiquary whose clients included Charles Dickens (a link with Sibson through his younger brother) who depicted him as Mr Undery in his weekly magazine *Household Words*.^11^ They had no children but it was a very happy marriage. They hosted regular breakfast parties for his students ‘where prayers might punctuate a discussion of a post-mortem’.^8^ He was popular with the students though ‘Sibby’, as he was generally known, was thought by some to be ‘a little too hot on the subjects of auscultation and percussion but still a really good fellow’.^6^ Although an immensely busy man and always very punctual, he could be disorganised in his working habits, leaving his papers scattered around the house and dependent on his wife to retrieve them.^2^

### The inner man

Even allowing for the fulsome nature of some 19th century obituaries the praise heaped on Sibson after his death was exceptional. Epithets used to describe his character included genial, warm-hearted, sympathetic and unselfish, always cheerful, a man of generous impulses and one of the truest of friends. However, like many of his generation, he was not sympathetic to the idea of women doctors and opposed admission of women to medical degrees in the University of London ‘as fervently as he led the opposition to a suggestion that St Mary's Hospital medical school admit women medical students in 1869’.^8^ In his work he was earnest, diligent, meticulous, upright and indefatigable. He abhorred self-seeking and unpunctuality.^2,6,8,12^ He never sought great influence in any institution but his natural aptitude for business, his quick marshalling of figures and his clear perception of the most appropriate course of action meant that he was always in demand.^2,6,8,12^ He was described as fair of complexion and as above medium height. The Wellcome Library holds an image of him but it is not used here because ‘there was not any portrait which in the judgment of his friends constituted a fair representation of his personal appearance’^2^ and none appeared in any obituary.

He was a member of the Alpine Club of London, founded in 1857 and the world's first mountaineering club.^13^ With others he made the first ascent of the Lyksamm (4532 m) on the Swiss–Italian border in 1861.^14^ His training for his annual autumn holiday in the mountains was unusual, certainly by modern standards. An early riser, he was out by 6–7 a.m. in all seasons, striding and running across Hyde Park and Regent's Park, sometimes with a brisk ascent of Primrose Hill, always with a 9 lb (4 kg) iron bar across his shoulders. Sometimes he would sit by the Serpentine lake, cigar in mouth, reading *The Times* newspaper. On cold mornings he might wear several coats, perhaps with a rug pinned over them.^2^

Less vigorous hobbies included an interest in Gothic Architecture, on which he published a (now lost) pamphlet, despite the concerns of Hodgkin that this might damage his medical career. He was also a knowledgeable collector of Wedgewood pottery, especially the work of the sculptor John Flaxman RA (1755–1826)^2^ who was also the first professor of sculpture at the Royal Academy. He corresponded with the artist James McNeill Whistler who he would have known through their membership of the Burlington Fine Arts Club.^15^

Charities with which he is known to have been involved included the Society for the Relief of Widows and Orphans of Medical Men, the Royal Medical Benevolent Foundation and Nurses for the Sick. The Society for the Relief of Widows and Orphans of Medical Men, now the Association for Assistance of Medical Families, was founded in 1788 in the Gray's Inn Coffee House. At first, the Society helped only those living in London and its immediate vicinity. In 1964 the by-laws were altered to permit doctors resident within 60 miles of Charing Cross to become members and the Society now provides its services nationwide.^16^ Sibson was a sometime director of the Society.^17^ Fifty-eight widows and 256 children were receiving assistance in 1870.^18^ In 1855 Dr John Propert (1793–1867), who was also a member of the Society for the Relief of Widows and Orphans of Medical Men, founded the Royal Medical Benevolent Foundation which is now almost synonymous with the school, Epsom College, but which originally included accommodation for impoverished medical practitioners and their close relatives. Sibson had attended a fund raising event in 1854 and was listed in the founding Act as one of the initial seven governors of the Foundation.^19^ The Metropolitan and National Nursing Association for Providing Trained Nurses for the Sick Poor was founded in 1875 by Florence Lees (1840–1922), the leading pioneer of district nursing.^20^ The association was funded by charitable donations but Sibson was keen that those who could ‘should be asked to contribute something, however small….as by so doing that spirit of independence so necessary for the welfare of the labouring classes, would be encouraged…’.^21^

### Death

Sibson died very suddenly at the Hotel des Bergues in Geneva as he was returning from his annual mountaineering holiday.^22^ He had experienced previous episodes of numbness and pain in his left arm and two episodes of syncope in preceding year^2^ and, perhaps because he had published on aortic aneurysms,^23,24^ it was suggested that this was the cause of his death. However this was speculation because there was no post-mortem examination. He was buried in the graveyard of Acton Church where his wife later joined him.^25^ His will was proved by his brothers-in-law for under £6000 (equivalent to under £713,000 in 2023).^22,26^ But the value of his ‘extensive and beautiful’ Wedgwood Collection and other objets d’arts which included old English furniture^27^ was in excess of £7000 when sold the following year.^28^ He left his entire estate to his wife.^22^

## Sibson and infectious diseases

1831–1832 was the year of the first cholera epidemic in the United Kingdom and in 1832 Sibson worked at cholera hospitals in Leith, Newhaven and Edinburgh. He later claimed that the first cholera patient to be treated with a saline injection had been under his care in Newhaven. She recovered as did the next two but then three out of the next four died and after further failures while working in Edinburgh he gave it up. Thirty four years later he stated that ‘If fluid could be found of the right sort, he had no doubt it would be better’.^29^ Even though Sibson had worked in Edinburgh during the epidemic it would seem that he was unaware of the work of Thomas Latta, a general practitioner in Leith who, in 1832, was working in the Drummond Street Cholera Hospital in Edinburgh and who is now recognised as the successful pioneer of treating cholera by saline injections, having based the content of his saline infusates on the experimental work of William O'Shaughnessy.^30^

By 1849 John Snow (1813–1858) had already accumulated evidence to suggest that cholera was a waterborne disease. However, when he presented his evidence in October 1849 to the Westminster Medical Society most members, including Sibson, disagreed with Snow and still held to the long established belief that it was transmitted via the atmosphere. Neither did Sibson accept Snow's opinion that the disease started in the intestinal mucosa, arguing that cholera could occur without an intermediary stage of diarrhoea.^31^ By 1855 Snow had collected more persuasive evidence^32^ and more people were coming round to his way of thinking but whether Sibson was one of them has not been discovered.

Between July and December 1846 the Nottingham General Hospital treated 899 fever patients (223 in- and 676 out-patients) whereas the usual average number of in-patients was only 43 such cases. Sibson published a paper in which he described this dramatic event and which was an early recognition in Britain of the distinction between typhoid and typhus.^33^ An earlier British recognition, to which Sibson referred, had been published by Alexander Patrick Stewart (1813–1883) who had studied the conditions at the Glasgow Fever Hospital from 1836–1838 and discussed them with Auguste François Chomel (1788–1858) when in Paris.^34,35^ In 1840 Stewart presented a paper titled ‘Some considerations on the nature and pathology of typhus and typhoid fever applied to the solution of the question of the identity or non-identity of the two diseases’, to the Parisian Medical Society. This was subsequently published in the *Edinburgh Medical and Surgical Journal* where he provided strong evidence that whereas ‘the effluvia from living bodies in close and unventilated localities could generate the poison of typhus, typhoid fever often appeared in country places and in the best aired houses’.^36^

In an extensive review of the literature Sibson cited the work of another French physician, Pierre Louis, and followed the example set by Louis of presenting a numerical analysis of his clinical data.^37^ He noted that the fever began during an exceptionally hot summer, that the great majority of cases occurred in streets and yards which were neither paved nor macadamised and in which there were many pools of offensive foetid water which was often polluted by decomposing vegetable or animal matter or both. Equally poor people from healthier streets were less likely to be affected. The disease was transmissible within the hospital, attacking staff as well as other patients, and some convalescent patients ‘relapsed’ after returning home. The clinical features, which included rose spots in some though not all patients, were identical with the ‘fièvre typhoide’ described by Louis except that splenomegaly was rare in the Nottingham cases.

Sibson described how typhoid was distinguished from typhus by the invariable involvement of Peyer's patches and the presence of mesenteric lymphadenopathy in those patients who had autopsies, by the nature of any rash and by the presence of diarrhoea rather than constipation. Typhoid was predominantly a condition of summer and autumn and typhus of winter but ‘through the influence of contagion the winter typhus is prolonged into summer and the autumn fever [typhoid] into winter’.

He considered that the ‘manifestly contagious’ nature of typhoid was due to a ‘poisonous effluvium’ that was

undoubtedly drawn into the lungs at each inspiration, and whether it consists of animaliculi, as Drs. CJ Williams and Henle suppose, or of particles of decomposed matter, like musk, too subtle to be detected by our present means of investigation, the effluvium certainly penetrates with the air, through the lungs into the blood to all the nerves and to every part of the frame.^33^

In 1847, therefore, Sibson could conceive of typhoid as a transmissible airborne effluvium (he does not use the word ‘miasma’) which contained either animate or inanimate matter and which was inhaled and distributed throughout the body; yet so ingrained was his belief in the long established theory of *air*borne transmission of miasmata as a cause of disease that two years later he was unable to accept Snow's *water*borne theory of the transmission of cholera.

The distinction between typhoid and typhus had originally been made by Gerhard in Philadelphia in 1837^38^ but his work and that of the young tyros Sibson and Stewart was not finally accepted in Britain until confirmed by William Jenner in London in 1849.^39^ The delay in making the distinction in the United Kingdom may also have been, as Sibson noted, because it was ‘unfortunate that the Registrar-General groups all fevers under the common head of typhus’, thereby combining typhoid, typhus and other fevers in a single category.^33^ It was not until 1868 that practitioners were asked to distinguish in their certificates between typhoid and typhus fever.^40^

Rabies (hydrophobia) is a fatal disease caused by a neurotropic RNA virus, *Rabies lyssavirus*, which is most commonly contracted through dog or fox bites. Charles Waterton (1782–1865) explorer, naturalist and pioneer conservationist, who brought wourali (curare) to Britain from Guiana, credited Professor William Sewell (1781/1782–1853) of the London Veterinary College with the suggestion that wourali might prove life-saving in rabies.^41^ Sibson and Waterton had discussed this possibility but Waterton was reluctant to distribute samples of wourali because he wanted to be present when it was used to treat a human case. When such a case did occur in Nottingham, which was 65 miles from Waterton's home near Wakefield, the patient had died before he arrived. Thereafter the two men collaborated on experiments with artificial respiration in two asses who had been poisoned with wourali, one of which survived. Waterton noted that Sibson ‘had most wonderfully improved the bellows, and thus rendered the process much less laborious’.^41^ Sibson gave only a brief description of these experiments before moving on to other projects but Hermann Beigel (1830–1879), physician to the Metropolitan Free Hospital in London, to whom Sibson lent his detailed notebooks, provided further information and credited Sibson with findings which were later observed and published by Claude Bernard.^42^

## Sibson's interest in ether and chloroform

Sibson's initial interest in volatile inhalational anaesthetic agents was not for the purpose of surgical anaesthesia but for the relief of trigeminal neuralgia where the object was to relieve pain but not necessarily to render the patient unconscious. His published results on this subject, like those of others, contained too few patients to provide meaningful results but in some there was worthwhile interruption of the pain cycle.^43,44^

It is evident from his other publications that Sibson had significant experience of surgical anaesthesia and on the subject of what he referred to as ‘narcotic poisons’ more generally.^45,46^ Sibson and John Snow cooperated on research on animals on at least two occasions^47^ and Sibson was present on at least one occasion when Snow was anaesthetising a patient.^48^ Snow credited Sibson with being the first to point out that deaths due to chloroform in humans were cardiac in origin^49^ but they did not always agree on other effects of chloroform in man.

For example, Sibson believed that most cases of instantaneous death attributed to chloroform were due to a combination of chloroform and fear because they typically occurred in minor rather than major operations and the patient had been almost more fearful of the anaesthetic than of the operation.^43^ He thought those most at risk were not so much those with organic heart disease but those in whom ‘palpitation and dyspnoea are easily excited, either from abdominal distension or from mental emotion’.^43^ Maltby observed that this was an opinion which Sibson had not supported with factual evidence. However he also noted that the ventricular fibrillation which Levy and Lewis had later, in 1911, shown to be the cause of such sudden deaths in cats lightly anaesthetised with chloroform was often preceded by ventricular extrasystoles.^25,50^

By contrast Snow insisted, on the basis of his experiments on animals, that sudden cardiac death due to chloroform was almost always consequent upon chloroform concentrations in excess of 5% as a consequence of the over-rapid absorption of chloroform when the vapour was administered on a handkerchief, sponge or an inadequate or faulty inhaler. He considered that fear had no part to play in deaths due to chloroform because it had already been abolished by anaesthesia.^51,52^ The necessity for chloroform to be diluted with at least 94–95% air was one to which Snow was to return to repeatedly over the years right up to the year of his death.^53^ He did however describe two cases where he attributed the cause of death to the evident presence of fear or to mental emotion in patients who he believed could not have inhaled sufficient chloroform to have had any biological effect. In one of these cases he had biochemical evidence of the absence of chloroform in blood, lungs and liver in samples obtained 24 hours after death.^54,55^ Sibson believed that, if possible, chloroform should not be administered in the sitting position because this posture required the heart to work harder than when recumbent.^45^ On the other hand Snow thought that the lying position was only necessary if the patient fainted and he cited 949 of his own cases in which he had administered chloroform uneventfully to sitting patients.^56^ The generally accepted figure for deaths attributable to chloroform in later years was 1:3000–1:6000^57^ so it is not surprising that a knowledgeable and cautious practitioner like Snow might have 949 cases without a death. Sibson also thought that chloroform should only be used for dental extractions in exceptional circumstances, but again Snow disagreed citing 867 uneventful cases of chloroform use in the dental chair.^58^ Attitudes to both risk and pain change over time. Many practitioners, especially in North America and later in the United Kingdom, came to regard the mortality associated with chloroform as too high in relation to that with ether (1:14,000–1:2800).^57^

Snow and Sibson would certainly have agreed that chloroform should never be administered solely under the supervision of the operating surgeon. As Sibson wrote: ‘This should never be. Chloroformisation is the exhibition of a subtle poison and ought to be watched by the administrator with undivided attention during the whole of its operation’.^45^

Snow had not thought that there were any significant pupillary changes under anaesthesia.^59^ However Sibson noted that under the

increasing influence of ether and chloroform the pupils first contract, then oscillate between contraction and dilatation, and finally dilate. So long as the pupil is contracted a dreamy state often exists, and the patient, when operated upon, frequently manifests an unremembered consciousness; he is, in fact, in the state of stupor. When the pupils dilate, and the iris is immovable, consciousness is extinguished, and the patient is in the state of coma.^60^

**Figure 1.**
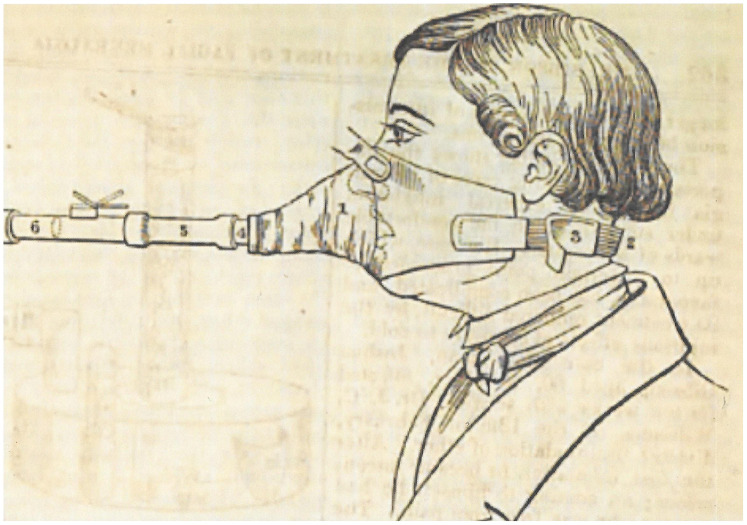
Sibson's anaesthetic face mask. From the *London Medical Gazette* 1847; n.s. 4: 358–364.

Although Zimmer wrote about a French version of an inhaler described by Sibson,^61^ what Sibson actually described was a mask made from Macintosh raincoat fabric and lined with oiled silk. Vulcanised India-rubber straps kept it in place by means of a buckle. Sibson used it in conjunction with Snow's Mark I ether vaporiser (Figure 1). The mask was more comfortable than Snow's original design, partly because the connection to the vaporiser was more flexible and partly because it covered both mouth and nose so that the operator did not have to pinch the nostrils.^43^ For this latter reason Snow described it as ‘one of the greatest mechanical aids to the process of inhalation which has been contrived in modern times’. Snow also noted that Dr Hawkesley invented a similar mask about the same time, in February 1847, but that he did not make it known.^62^ Snow soon adopted Sibson's mask but, as Duncum observed, he seldom touched a piece of equipment without improving it further. In this instance he made it even more adaptable to faces of different shapes as well as incorporating valves.^63,64^

**Figure 2.**
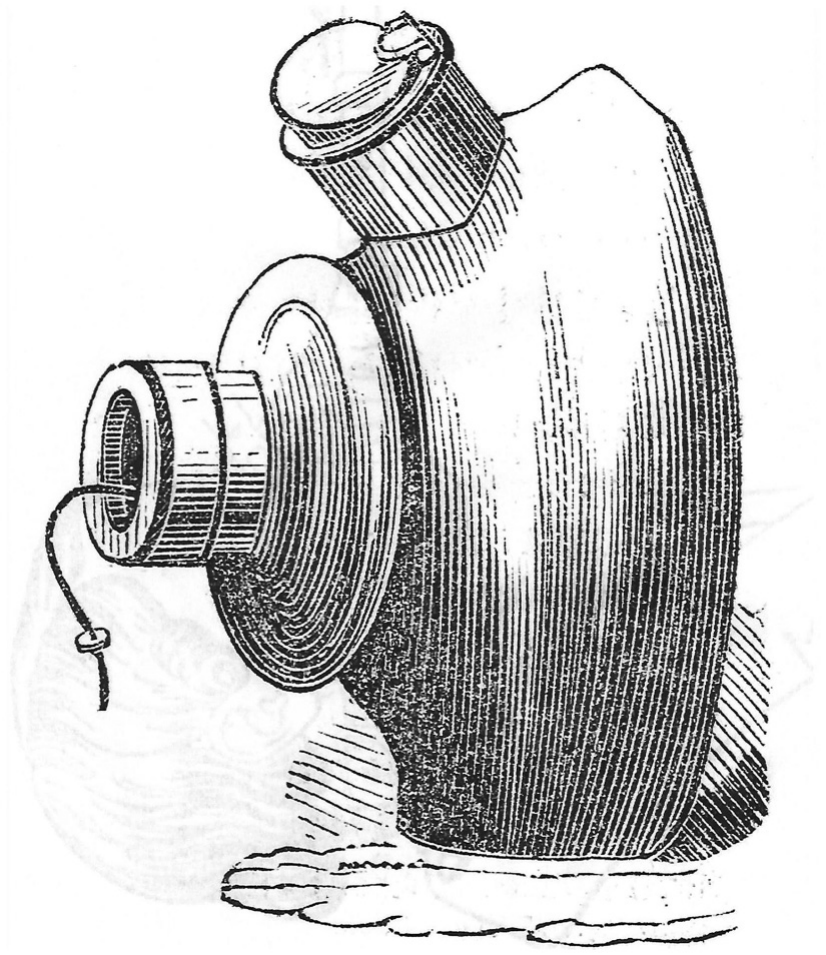
Sibson's chloroform vaporiser. From the *London Medical Gazette* 1848; n.s. 6: 267–271.

Sibson also designed a mask for chloroform which presumably incorporated a sponge for chloroform inside it (Figure 2). He believed that if respiration stopped during the administration of chloroform then artificial respiration must be started immediately as all other suggested remedies had proved useless. To this end his chloroform mask could be instantly adjusted to facilitate artificial respiration because the upper (expiratory) valve could be easily removed to allow ingress of air while the mask was held in place.^61^

## An inventor of medical equipment

**Figure 3.**
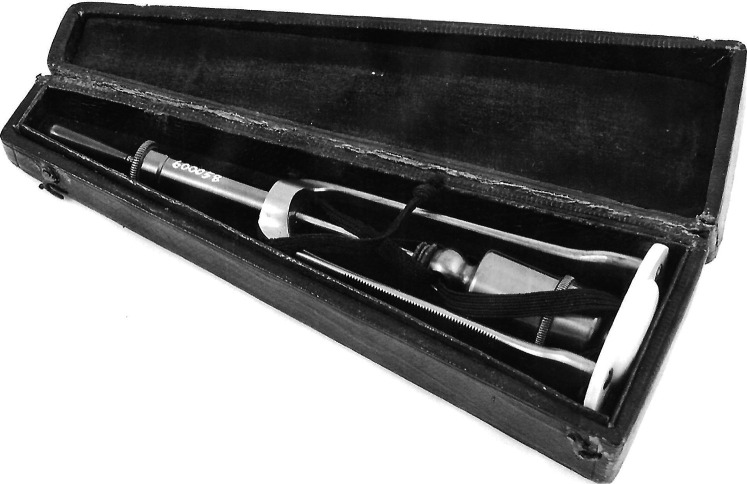
Sibson's combined percussor and pleximeter. Courtesy of the Science Museum Group. https://creativecommons.org/licenses/by-nc-sa/4.0/.

**Figure 4.**
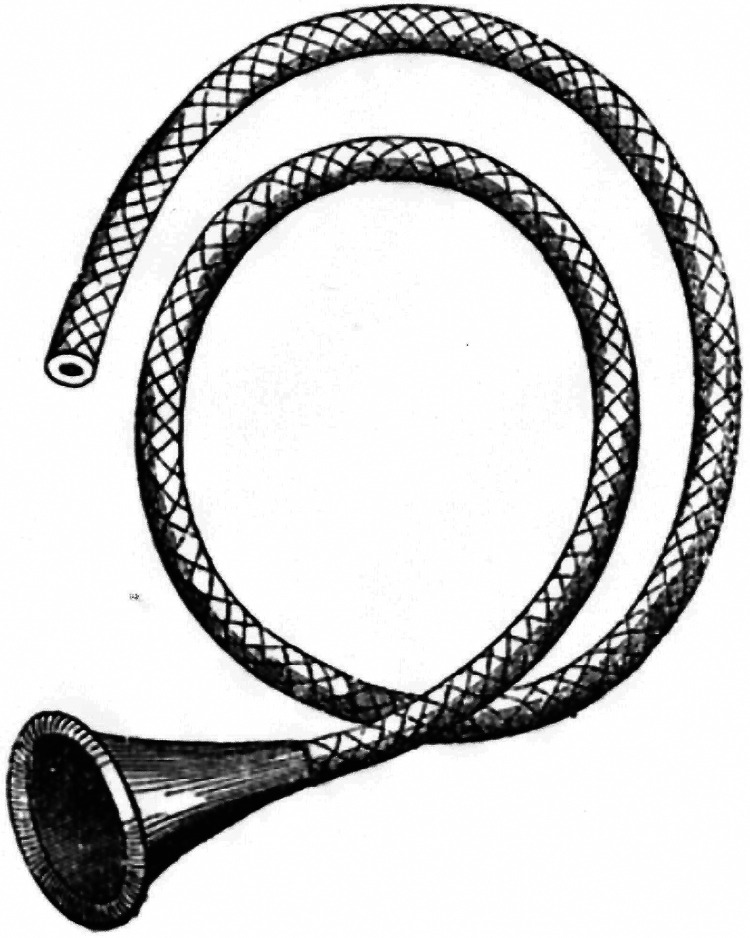
Sibson's flexible monaural stethoscope. From the *London Medical Gazette*; 1841; n.s. 2: 911–912.

Anaesthetic equipment was not the only apparatus invented by Sibson. Like many of his contemporaries he was a compulsive inventor of new equipment, although some of his inventions were minor modifications of those designed by others. Joseph Auenbrugger (1722–1809) had invented the clinical practice of percussion, allegedly having learned its use in the cellar of his father's inn where it was used to assess the residual contents of wine casks.^65^ Sibson's combined percussor and pleximeter (Figure 3) was designed to give clearer sounds than using the fingers. It also standardised the force of the percussive strike.^66^ He introduced a chest-measurer for ascertaining the diameter of the chest. It was capable of accurately measuring the movements of respiration to the hundredth part of an inch.^67^ Another invention was a flexible monaural stethoscope^68^ (Figure 4). Others had already designed such an instrument but with an earpiece which had to be held against the ear. Sibson simply removed the earpiece so that the tubing could be inserted into the user's aural canal. This left a hand free to monitor the pulse while listening to the heart. He also noted that the intensity and quality of some perceived sounds varied with the amount of pressure which was applied by the bell of the stethoscope on the chest wall. Increasing the pressure would have had the effect of using the patient's skin as a diaphragm. He designed a cardiagraph [*sic*] to obtain measurements of the surface impulse of the heartbeat, analogous to an arterial sphygmograph.^69^

## Medical anatomy and cardio-respiratory research

The work which Sibson might have regarded as his *opum magnus*, because it had involved him in many hours of meticulous dissection and because it was produced in instalments from 1855 to 1869, was his book on *Medical Anatomy*.^70^ This work, which was aimed at physicians rather than surgeons, demonstrated the relative positions of the chest and abdominal organs in varying physiological positions and in disease. Sibson wrote the commentaries on the detailed lithographic illustrations made by the artist and lithographer William Fairland (b. 1806). Fairland had previously done lithographical work for John Flaxman which may have been how Sibson came to know of him. A review in the *Lancet* praised the quality of the illustrations and the laborious work which constituted the basis for Sibson's commentaries on the plates but had less to say about the value of the work and it does not appear to have been cited often.^71^ Sibson's interest in the subject of changes in the relative anatomical positions of different organs can be traced back to at least eleven years before the first fascicule of *Medical Anatomy*^72^ and, as mentioned earlier, may have been triggered by the influence of Lizars. His interest in the subject also continued after *Medical Anatomy* had been published and was evidently a life-long passion.^73^ Another long-standing interest was in pericarditis.^74,75^ He also published chapters on adherent pericardium, endocarditis, the position and form of the heart and great vessels in *A System of Medicine* by Russell Reynolds,^75^ and, as already mentioned, papers on aortic dissection; and the first of his two Lumleian Lectures concerned the effects of Bright's Disease on the heart.^76^

## Vivisection and the antivivisection movement 1875–1876

Aware that British research in medicine and physiology in the early 1870s was lagging behind that of colleagues in mainland Europe, there was a conscious effort to emulate the work of those colleagues with a consequent rapid increase in experimentation on live animals. Much of this research involved surgical procedures for which anaesthesia was not always used. This led to an increasingly vocal anti-vivisection movement. This campaign was not confined to the laity as some eminent doctors eschewed scientific research on animals, preferring to view the practice of medicine as an art which was focussed entirely on the patient and to which animal research was irrelevant.^77^ In 1875 the concerns led to the appointment of a Royal Commission which examined 53 witnesses.^78^ Sibson was one of those called to give evidence, his name probably having been proposed by proponents of animal experimentation which, as mentioned, he had practised and because he had a reputation as a sound performer on committees. In the event his answers were not as satisfactory as his backers might have hoped. He tended to minimise any pain experienced by the animals and, even though it was already clear that some legislation would be required, if only to placate the antivivisection lobby,^79^ he argued that rules to govern animal experimentation were unnecessary and could be unduly restrictive. Instead he maintained that it would be much better to leave decisions to the individual experimenter, each governed by his own conscience and sense of what was right and by public opinion. His questioners’ responses suggest that they thought he was out of touch with both reality and public opinion. Equally damaging to his testimony was his clash with TH Huxley (1825–1895) on the disputed question of whether curare possessed anaesthetic properties. Claude Bernard (1813–1878) had claimed that curare affected only the motor nerves but many British physiologists, including Thomas Lauder Brunton (1844–1916), believed that it also affected sensory nerves.^77^ Rather than admitting that this was a contentious area, Sibson claimed that 8–10 minutes after administration of curare there might still be reflex activity but consciousness and sensibility would have gradually faded away and there would be no sensation of feeling whatsoever. However he did admit that this was his opinion and he did not provide any evidence to support it.^78^

In the event Sibson's evidence was overshadowed by the extraordinary responses of another witness, the histologist and bacteriologist, Edward Emanuel Klein, F.R.S. (1844–1925). Klein admitted that, except when he was teaching ‘where one must take regard for the feelings and opinions of those before whom one does the experiment’, he only used anaesthetics for his own convenience and that he had ‘No regard at all’ for the suffering of the animals. Before coming to England Klein had practised in Vienna and thought that it was the general practice on the Continent to disregard the feelings of the animal. Under pressure from colleagues Klein subsequently tried, unsuccessfully, to amend his evidence but it was now inevitable that legislation would be brought in.^78^

Sibson was also one of those who lobbied Lord Carnarvon when he introduced the Cruelty to Animals Bill in 1876. As part of the antivivisection movement this Bill, if enacted, would have placed considerable restrictions on animal experimentation. Sibson cited evidence that the Act would effectively prevent the practice and teaching of modern physiology, thereby lowering the standard of medical practice and the standing of the profession in the United Kingdom.^80^ However the Bill was enacted later that year. Under the Act those wishing to carry out experiments on live vertebrates required a licence. Experiments could be conducted on un-anaesthetised animals if it could be shown that this was necessary to the aims of the experiment. Curare was not defined as an anaesthetic but debate on this matter continued for several decades.^81^ Penalties for infringing the Act were a fine of up to £50 for a first offence and up to £100 or three months imprisonment for any subsequent offences.^82^

## Medical societies and other medico-political interests

Sibson's medical interests extended beyond clinical and scientific matters. He was actively involved in many medical institutions and societies and membership of some of these led to medico-political activities.

At the *Royal College of Physicians* he was an Examiner, Censor, Goulstonian (1854), Croonian (1870) and Lumleian (1874) Lecturer, and Curator of the Pathological Museum. Undoubtedly his most important work at the College was as principal instigator and secretary to the Committee on the Nomenclature of Diseases,^83^ a work of ‘enormous labour’ as it was described by Sir James Paget and others.^2,6,12^ The many and lengthy trials and tribulations involved in bringing it to fruition have been described by Robb-Smith. It was initially proposed that Dr Sibson should receive an honorarium of 100 guineas for his long and very valuable service in connection with the *Nomenclature* but in the event the College presented him with a large classical silver vase on a silver pedestal.^40^ This work listed English terms for diseases alongside their Latin, French, German and Italian equivalents and remained the standard work on the subject until the publication of the first edition of the World Health Organization's *Manual of the international statistical classification of diseases, injuries and causes of death* in 1960.

As a Council member of the *Royal Society* his fellow members included the President, the botanist and explorer, Sir Joseph Hooker, the biologist and anthropologist Thomas Henry Huxley, the anatomist and surgeon Sir Benjamin Brodie and the physiologist Sir John Burdon Sanderson. He was also joint treasurer, with his friend Sir Douglas Galton, of the Royal Society Club, a dining club for Fellows.^2,6^

At *The Royal Medical and Chirurgical Society* (the predecessor of the Royal Society of Medicine) he was a member of the committee which produced the significant, if somewhat controversial, report on chloroform in 1864.^84^ At the *Harveian Society* he was president in 1855 and Harveian Lecturer in 1875^85^ and in the *University of London* he was an examiner in medicine from 1863 to 1865 and a member of Senate in 1865–1868.^2,6^ Other societies with which he was associated included the *Medical Society of London*, the *Clinical Society of London*, the *Westminster Medical Society*, the *Epidemiological Society* and the *Medical Teachers Society*.

His longest association was with *The British Medical Association* (BMA), of which his membership dated back to 1843 when it was still the *Provincial Medical and Surgical Association* (PMSA, founded 1832) and in which he succeeded the founder, Sir Charles Hastings, as President of Council in 1866. He was the instigator and first chairman of the Scientific Grants Committee from 1874 until his death and became associated with several medico-political issues.^2,6,8^ These included the conditions of service of Poor Law Medical Officers, sale of arsenic, the conditions of service of army medical officers and the direct representation by members of the profession on the General Council of Medical Education.

Complaints about the conditions of service of Poor Law Medical Officers were an almost constant theme in the 19th century. In 1846–1847 Sibson was a member of a PMSA committee which lobbied the Home Secretary and the President of the Poor Law Commission on terms and conditions of service but without success.^86^ When new duties were imposed upon Poor Law Officers without additional payment in 1849 Sibson put forward a motion at the PMSA in an attempt to rectify this situation.^87^

In 1837–1838 there were 185 recognised cases of death by arsenic poisoning, representing more than one-third of all deaths due to poisoning.^88^ Concerns rose as numbers increased during the 1840s, perhaps in part due to improved recognition and reporting. At this time arsenic could be bought freely and was often used in wallpapers and sheep dips. Together with Dr Jonathan Toogood (1784–1870), founder of the Bridgwater Infirmary and others including the Pharmaceutical Society, Sibson lobbied for restrictions to be placed on its sale, hoping that this could be limited to doctors and pharmacists.^88,89^ Their efforts led to the Sale of Arsenic Regulation Act 1851 (14 & 15 Vict. c. 13). This had little success in limiting sales because it could not specify that sales be limited to doctors and pharmacists as these professions were still not legally defined. However it did contribute to the case for medical reform and it also demonstrated a clear sense of social responsibility by the Provincial Medical and Surgical Society.^90^

Before 1858 medical officers in the British Army did not enjoy equal status with combatant officers with regards to pay, status or conditions of service, but most of these deficiencies were remedied by a Warrant of 1858. This followed a petition to both Houses of Parliament by the Metropolitan Branch of the BMA in 1855. However by 1864 many of the benefits in the 1858 had already been reversed.^90^ Once again the Metropolitan Counties Branch was to the forefront and a deputation led by Sibson, its president at this time, had an interview with His Royal Highness Prince George, the Duke of Cambridge, a career soldier who had been promoted to Commander in Chief of the Army, a promotion which may have owed more to his status than to his ability. Sibson's deputation sought the re-instatement of all aspects of the 1858 Warrant but the Duke, who was famously opposed to reform, conceded nothing.^91^ However two days later a second deputation, again led by Sibson, had rather more success with Dr Gibson, the Director-General of the Army Medical Department, who would have been more acutely aware of the consequences of the failure to recruit army medical officers at that time.^92^ Even so it was not until 1876 that most of the hard-won concessions in the 1858 Warrant were restored.^90^

When the General Council of Medical Education (now the General Medical Council) was created by the Medical Act of 1858 it consisted of a president, six members nominated by the Crown and 17 members nominated by those universities and medical corporations which awarded medical degrees and diplomas. As one of the roles of the Council was to ensure that curriculae and examinations set by these bodies were appropriate and properly monitored, there was an obvious conflict of interest for 17 of the Council's members. Moreover the great majority of the medical profession, whose registration fees were the Council's sole source of income, had no representation on the Council. Sibson was prominent among those who thought both of these points to be wrong and he made full use of his presidency of the BMA to lobby in support of what became known as ‘Direct Representation’.^93^ He led a deputation to the Council which, with its inbuilt majority of vested interests, declined to lobby the government for a change in its constitution.^94^ It was not until 1886 that direct representation was finally achieved.^90^

It is apparent from these examples that Sibson's medico-political activities were always on the side of the under-dog, of the oppressed and even when unsuccessful they laid the groundwork for success by others at a later date.

Other public work which he was asked to take on by the Government included membership of a committee enquiring into the state of the Greenwich Hospital (a hospital for naval pensioners), membership of the Cubic Space Working Party of the Poor Law Board which specified space requirements for inmates of Poor Law Infirmaries (a working party on which his friend Douglas Galton played a major role) and membership of the Metropolitan District Asylums Board. He was also consulted on the choice of sites for infectious diseases hospitals where, like Galton and Florence Nightingale, he spoke in favour of pavilion designs of no more than two storeys.^2,8,95^

## Conclusion

Sibson is little known today but was a major figure in British medicine in the mid-19th century. He had to make his own way in the world and did so with great success. He was immensely energetic and capable and his interests were many and wide-ranging. He was well liked and respected as a clinician and as a researcher. He was also much in demand as an office holder in his societies and as an expert opinion on committees and enquiries.

What was unusual about Sibson's career was not only that he practised first as a surgeon before practising as a physician but that, while practising as a physician, his areas of special interest were chronologically quite separate: first, infectious diseases, then pain relief with volatile anaesthetics and finally cardiorespiratory diseases, while all the time maintaining his early interest in clinical anatomy. Such people are most unusual. Another example is Arthur Ernest Sansom (1839–1907) who practised, albeit always primarily as a physician, but also initially as an anaesthetist, then as a specialist in infectious diseases and finally as a cardiologist.^96^ Sibson's biographer, William Miller Ord, practised first as a general practitioner, then as a surgeon at which time he also published on epidemiology and finally as a physician. Like Sibson he lectured in anatomy but in his case while he was a surgeon. As a physician he lectured in physiology.^97,98^

As Sir James Paget said of Sibson after his death: ‘He was a many-sided man, and on all sides good’.^12^ Had he lived longer and had he been less self-effacing, perhaps he might have received a national honour in acknowledgement of his considerable contributions to life in 19th-century Great Britain. He certainly deserves better recognition now.
